# Wants to Know

**Published:** 1867

**Authors:** 


*Wants to Know.*—The *Lancet*, noticing the Editorial change in the JOURNAL, is profoundly impressed with the importance of the question: "Has this change any significance?"

With uplifted castor and blandest smile, the JOURNAL is happy to answer—IT HAS.

